# Hypokalemic Paralysis and Hypocalcemic Tetany: Paradoxical Duality in a Case of Sjogren's Syndrome

**DOI:** 10.7759/cureus.48268

**Published:** 2023-11-04

**Authors:** Abhinav Kadam, Sourya Acharya, Sunil Kumar, Samarth Shukla, Rucha Sawant

**Affiliations:** 1 Department of Medicine, Jawaharlal Nehru Medical College, Datta Meghe Institute of Higher Education and Research, Wardha, IND; 2 Department of Pathology, Jawaharlal Nehru Medical College, Datta Meghe Institute of Higher Education and Research, Wardha, IND

**Keywords:** sjogren's syndrome, distal tubular dysfunction, albuminuria, aminoaciduria, acidosis

## Abstract

A 26-year-old female was hospitalized with acute lower motor neuron quadriplegia. Laboratory tests pointed to the presence of distal renal tubular acidosis, which was characterized by hyperchloremic metabolic acidosis, severe hypokalemia, alkaline urine, and a positive urinary anion gap. She also had aminoaciduria, hyperphosphaturia, hypophosphatemia, and normoglycemic glycosuria, all of which are indicative of dysfunction of proximal tubules. Further investigation confirmed Sjogren's syndrome. Strangely, our patient also experienced carpopedal spasms and had low calcium and magnesium levels. As the hypokalemia, hypocalcemia, and acidosis were corrected, the quadriplegia and carpopedal spasm improved. By the time of discharge, proximal tubular abnormalities were rectified (with the exception of albuminuria). One well-known renal symptom of Sjogren's syndrome is distal tubular acidosis. The brief proximal tubular dysfunction and distal tubular acidosis in Sjogren’s syndrome is rare. This case report highlights a rare renal complication of Sjogren’s syndrome.

## Introduction

Sjogren's syndrome is a multisystemic autoimmune condition that mostly involves exocrine glands. Despite the fact that it mostly affects the salivary and lacrimal glands, in 33% of the patients, additional glandular manifestations have been recorded [1,2]. A typical extra glandular feature is autoimmune tubulointerstitial nephritis, which can manifest as distal renal tubular acidosis (RTA) and/or nephrogenic diabetes insipidus in up to 25-35% of patients. Abnormalities of the proximal tubule are less frequently observed symptoms [1,3,4]. RTA in Sjogren's syndrome usually causes hypokalemia. Rarely Sjogren's syndrome can present as Fanconi's syndrome due to impairment of reabsorption in the proximal convoluted tubule. When associated with tubulointerstitial nephritis, it can result in hypophosphatemia and hypomagnesemia. Metabolic acidosis can occur due to both proximal and distal tubular dysfunction.

## Case presentation

**Figure 1:**
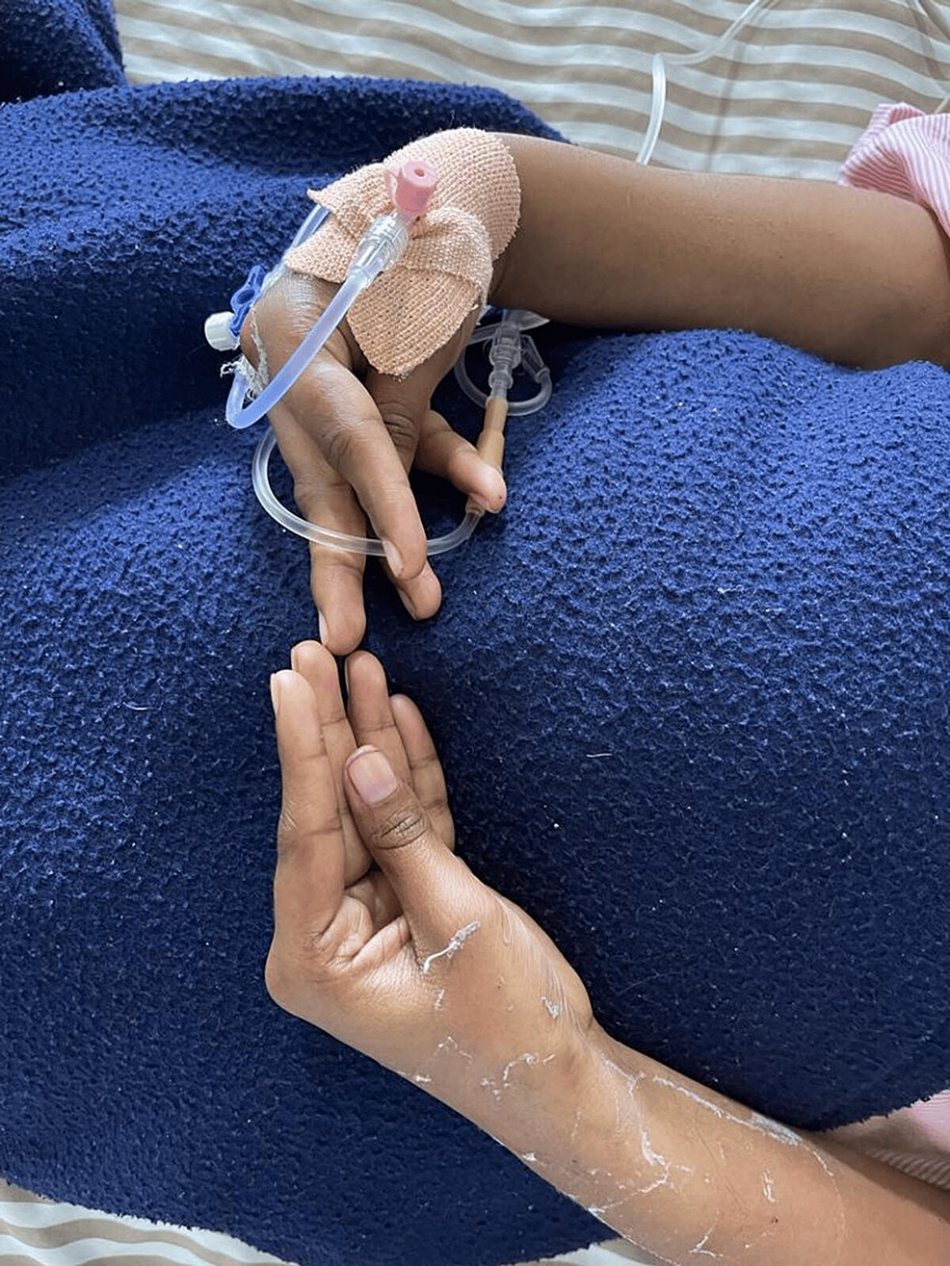
Photograph showing carpopedal spasm

A 26-year-old female presented to the hospital with profound weakness in all four limbs, which was insidious in onset, non-progressive, and continuous with no specific aggravating or relieving factors. On clinical examination, the patient was conscious and oriented, and obeyed verbal commands. She was afebrile and vitally stable with a pulse rate of 88 beats/min and blood pressure of 130/70 mmHg. General examination did not reveal any abnormality. Urine output was found to be within normal limits. Central nervous system examination revealed features of lower motor neuron lesion, such as areflexic flaccid quadriparesis without sensory or cranial nerve involvement with bilateral flexor plantar reflexes. Within the second hour of admission, she experienced an episode of carpopedal spasm (Figure 1), which improved after IV calcium gluconate and IV magnesium sulfate administration.

Her renal and thyroid function tests, complete blood count, blood sugar, and glycosylated hemoglobin (HbA1C) were found to be within normal reference ranges. Her parathyroid hormone assay (126.94 pg/mL; normal range: 13.6-85.8 pg/mL was found to be elevated. Her blood investigations are shown in Table 1. 

**Table 1:** Laboratory parameters of the patient along with their reference ranges. SGOT, serum glutamic oxaloacetic transaminase; SGPT, serum glutamate pyruvate transaminase

Laboratory parameter	Value	Reference range
SGOT	29 U/L	15-46 U/L
SGPT	27 IU	<35 U/L
Ionized calcium	1.21 mg/dL	4.4-5.6 mg/dL
Total calcium	8.4 mg/dL	8.4-10.2 mg/dL
Sodium	142 mmol/L	137-145 mmol/L
Potassium	1.30 mmol/L	3.5-5.1 mmol/L
Bicarbonate	9.5 mEq/L	22-30 mEq/L
Chloride	117 mmol/L	98-107 mmol/L
Magnesium	1.4 mg/dL	1.6-2.3 mg/dL
Anion gap	10 mEq/L	3-11 mEq/L
Phosphorus	1.8 mg/dL	2.5-6.5 mg/dL
Uric acid	2.2 mg/dL	2.5-6.5 mg/dL

**Figure 2:**
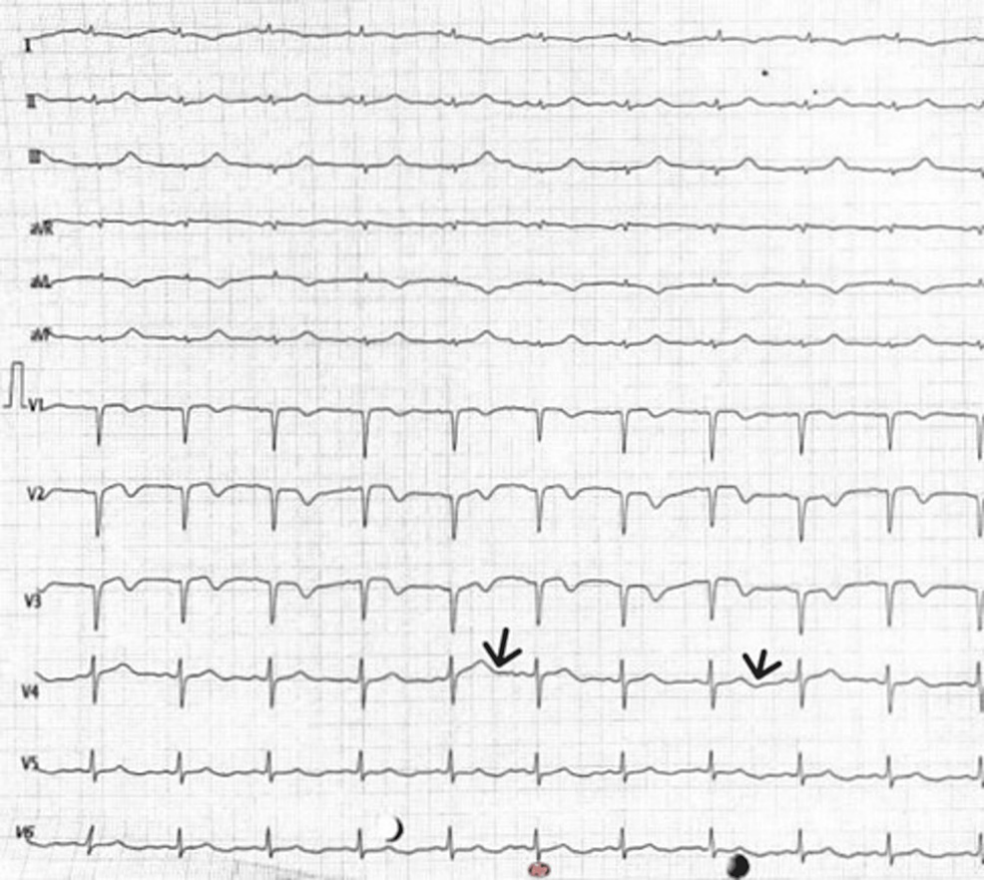
ECG showing ‘U’ waves

An evaluation of her arterial blood gas revealed compensated metabolic acidosis. Urine examination revealed albuminuria (1+), glycosuria (3+), and a pH of 6.2. Serum albumin was found to be 3.6 mg/dL, which was within the normal reference range. Her urine osmolality (294 mosm/kg) and urine spot sodium (149 mmol/L) were both normal, and her urine anion gap (+5 mEq/L; spot urine Na+ K- Cl) was positive. She exhibited a sinus rhythm with observable "U" waves on electrocardiogram (ECG) upon admission (Figure 2). MRI of the spine was normal.

Hypokalemia (1.30 mmol/L; normal range: 3.5-5.1 mmol/L)], hypophosphatemia (1.8 mg/dL; normal range: 2.5-6.5 mg/dL), hyperchloremia (117 mmol/L; normal range: 98-107 mmol/L) normal anion gap metabolic acidosis (10 mEq/L; normal range: 3-11 mEq/L), and hypouricemia (2.2 mg/dL; normal range: 2.5-6.5 mg/dL) were all discovered after analysis. Low osmolal gap (110 mosm/kg), positive anion gap in urine (10 mEq/L in metabolic acidosis), urine pH > 5.5, and normal urine salt levels all pointed toward a malfunction in the distal urinary acidification mechanism. Hypokalemia that has a transtubular potassium gradient (TTKG= [K+ Urine/K+ Plasma] / [Urine Osmolality/Plasma Osmolality]) of 20.4 mmol/L is a sign of tubular wasting. The existence of distal RTA was confirmed by these findings. However, signs of proximal tubular dysfunction were also evident, as seen by glycosuria associated with normal glucose level, aminoaciduria, albuminuria, hyperuricaciduria (fractional excretion of uric acid [FEUA]: 37.6%; normal: 10-12%), and hyperphosphaturia (fractional excretion of phosphate [FEP]: 24%; normal: 5-12%).

She had symptoms of Sicca’s syndrome, including a history of parotid swelling episodes that had occurred repeatedly, a dry mouth, and dry eyes. Schirmer's test results (2 mm in 5 minutes; normal range: 10-30 mm in 5 minutes), with elevated titers of anti-nuclear (1:80 titers, speckled pattern by immunofluorescent staining), anti-La, and anti-Ro antibodies discovered during additional testing supported the diagnosis of Sjogren's syndrome. Enzyme-linked immunosorbent assays for the human immunodeficiency virus and viral markers for hepatitis B and C came out negative.

The patient received bicarbonate replacement and IV potassium (160 mEq in 36 hours at the rate of 10 mEq per hour). Injection calcium gluconate was also administered as 10 mEq dissolved in 10 mL of normal saline over 10 minutes 8 hourly. She was not administered any oral phosphorus supplements. By the second day of admission, her serum potassium level had returned to normal, and she had fully recovered neurologically. Since the third day of her admission, she had no carpopedal spasms, and the "U" waves in her ECG stopped appearing. (Figure 2). By the third day, glycosuria was absent, but albuminuria continued to persist. By day 3, the amount of serum phosphate had increased, and by day 5, the level of uric acid had returned to normal. On the fifth day of hospitalization, measured fractional excretion of phosphate (10%) as well as uric acid (12%) reverted to normal range. Her urine pH was 7.0 and her serum bicarbonate was 16 mEq/L at the time of discharge.

On the day of discharge, oral prednisolone (40 mg once a day) was started. She was also instructed to keep taking potassium citrate and sodium bicarbonate. She had normal serum potassium, phosphate, and uric acid at the time of follow-up (two months later) but low bicarbonate (15 mEq/L). Her urine analysis revealed a pH of 6.6 and normal levels of FEP and FEUA. She had neither albuminuria nor glycosuria. One year after her first presentation, she was sustaining serum potassium level of more than 3.8 mEq/L and serum bicarbonate between 16 and 18 mEq/L, and her ocular problems subsided with the use of artificial tears.

## Discussion

Sjogren's syndrome is a chronic autoimmune disease that occurs due to the infiltration of lymphocytes into exocrine glands, which is best characterized by xerostomia and dry eyes. It may develop as the primary disease or as a result of another autoimmune condition. Our patient was confirmed to have primary Sjogren's syndrome in accordance with the updated international diagnostic criteria (American European Consensus group). Distal RTA and nephrogenic diabetic insipidus are the most typical renal manifestations [5]. With Sjogren’s syndrome, proximal tubular dysfunction is infrequent. There have been only 10 reports of generalized proximal tubular dysfunction associated with Sjogren’s syndrome to date. Except for one patient, they were all suffering from concomitant distal RTA, concentration problems, and renal impairment [6]. Along with proximal tubular dysfunction, she also exhibited distal tubular acidosis. Whether tubular dysfunction in hypokalemic nephropathy is permanent or reversible depends on the extent and duration of the hypokalemic episode as well as the pathological changes that result in the tubules [7].

While chronic hypokalemia will result in tubule atrophy, severe acute hypokalemia may cause intrarenal hypoxia, which may encourage tubule shrinkage or vacuolar degeneration without signs of damage. Hence, transitory proximal tubular dysfunction in our patient could be caused by either acute tubulitis or transient hypokalemic nephropathy.

Hypocalcemic tetany was another significant clinical sign of our patient. She experienced tetany (carpopedal spasm) within one to two hours of being admitted. She developed metabolic acidosis, as well as reduced serum ionized calcium and magnesium. Correction of the hypokalemia, hypocalcemia, and hypomagnesemia reduced carpopedal spasm. Patients with the illness known as hypocalcemic tetany experience carpopedal spasms as a result of low serum concentrations of calcium and magnesium ions. Tetany, a sign of neuromuscular excitability in a hypokalemic patient, is contradictory given that the most distinctive symptom of hypokalemia is flaccid paralysis [8,9]. Flaccid paralysis results from hypokalemia when it mostly affects the muscle, and carpopedal spasm occurs when it primarily affects the nervous system. Familial hypokalemic periodic paralysis (FPP) is a significant additional cause of flaccid quadriplegia in hypokalemic patients [10]. As sodium bicarbonate administered in RTA exasperates hypokalemic periodic paralysis and as acetazolamide used in FPP aggravates RTA, it is important to distinguish between the two [11].

Uncertainty exists regarding the precise mechanism via which Sjogren's syndrome induces distal RTA. One of the hypothesized explanations outlined in some investigations is the total lack of H-ATPase pump in the cortical collecting duct [4,5]. Autoantibody-induced inhibition of carbonic anhydrase 2, which leads in aberrant H+ secretion, is another proposed explanation [6]. In other case reports, the initial SS presentation was reported as hypokalemic paralysis [7-11].

Treatment should be concentrated on potassium correction rather than metabolic acidosis in cases of life-endangering hypokalemic paralysis. Alkali treatment may also worsen hypokalemia by causing bicarbonaturia and potassium to enter into the cells. Corticosteroids and other immunosuppressants are reserved for rapidly declining renal function [3]. Although effective therapy was documented in other case reports, one prior report had described the recurrence of RTA within six months of steroid therapy [11].

## Conclusions

Sjogren's syndrome may manifest as concomitant distal RTA with a proximal tubular dysfunction though the hallmark is distal RTA; however, a rare occurrence of distal RTA and temporary proximal tubular dysfunction with recovering renal function without the use of immunosuppressant medication has not been documented before. Sjogren’s syndrome should be ruled out in patients who present with normal anion gap metabolic acidosis. Proximal tubular acidosis is a rare complication of Sjogren’s syndrome and may present with Fanconi's syndrome. Such diffuse, distal and proximal, tubular dysfunction may occur in patients Sjogren's syndrome rarely. One likely explanation of proximal tubular dysfunction in Sjogren's syndrome is lymphocytic infiltration of proximal tubular cells and/or alteration of sodium-dependent apical transports.
